# The clinical course of comorbid substance use disorder and attention deficit/hyperactivity disorder: protocol and clinical characteristics of the INCAS study

**DOI:** 10.1186/s12888-022-04259-6

**Published:** 2022-09-23

**Authors:** Christoffer Brynte, Myriam Aeschlimann, Csaba Barta, Alex Hendikus Abraham Begeman, Amanda Bäcker, Cleo Lina Crunelle, Constanza Daigre, Laura De Fuentes-Merillas, Zsolt Demetrovics, Geert Dom, Lara Grau López, Romain Icick, Brian Johnson, Peter Joostens, Máté Kapitány-Fövény, Emily Karsinti, Falk Kiefer, Maija Konstenius, Frances R. Levin, Mathias Luderer, Wiebren Markus, Frieda Matthys, Franz Moggi, Raul Felipe Palma-Alvarez, Maria Paraskevopoulou, J. Antoni Ramos-Quiroga, Arnt Schellekens, Leila M. Soravia, Norman Therribout, Anil Thomas, Geurt van de Glind, Michiel Willem van Kernebeek, Sabine Vollstädt-Klein, Florence Vorspan, Wim van den Brink, Johan Franck

**Affiliations:** 1 Department of Clinical Neuroscience, Centre for Psychiatry Research, Karolinska Institutet and Stockholm Health Care Services, Region Stockholm, Sweden; 2 University Hospital of Psychiatry and Psychotherapy, University of Bern, Bern, Switzerland; 3 Department of Molecular Biology, Institute of Biochemistry and Molecular Biology, Semmelweis University, Budapest, Hungary; 4 De Hoop Ggz, Provincialeweg 70, 3328KP Dordrecht, The Netherlands; 5 Department of Psychiatry, Vrije Universiteit Brussel (VUB), Universitair Ziekenhuis Brussel, 1090 Jette, Belgium; 6 Department of Psychiatry, Addiction and Dual Diagnosis Section, Hospital Universitari Vall d’Hebron, Barcelona, Spain; 7 Psychiatry Group, Mental Health and Addiction, Vall d’Hebron Research Institute (VHIR), Barcelona, Spain; 8 Biomedical Network Research Centre On Mental Health (CIBERSAM), Madrid, Spain; 9 Department of Psychiatry and Forensic Medicine, Autonomous University of Barcelona, Barcelona, Spain; 10 Novadic-Kentron Addiction Care; Nijmegen Institute for Scientist Practitioners in Addiction, Nijmegen, the Netherlands; 11 Institute of Psychology, ELTE Eötvös Loránd University, Budapest, Hungary; 12 Centre of Excellence in Responsible Gaming, University of Gibraltar, Gibraltar, Gibraltar; 13 University of Antwerp, Antwerp, Belgium; 14 PC Multiversum, Boechout, Belgium; 15 Université de Paris Cité, Paris, France; 16 SUNY Upstate University, Syracuse, USA; 17 Alexianen Zorggroep Tienen, Tienen, Belgium; 18 Faculty of Health Sciences, Semmelweis University, Budapest, Hungary; 19 National Institute of Mental Health, Neurology and Neurosurgery – Nyírő Gyula Hospital, Budapest, Hungary; 20 Université Paris-Nanterre, CliPsyD Laboratory FR and APHP, Fernand Widal Hospital, Universitary Department of Psychiatry and Addictology, Paris, France; 21 Department of Addictive Behavior and Addiction Medicine, Central Institute of Mental Health, Medical Faculty Mannheim, University of Heidelberg, Mannheim, Germany; 22 Mannheim Center for Translational Neurosciences (MCTN), Medical Faculty Mannheim, University of Heidelberg, Mannheim, Germany; 23 Vagelos College of Physicians and Surgeons, New York City, USA; 24 New York State Psychiatric Institute, New York City, USA; 25 Department of Psychiatry, Psychosomatic Medicine and Psychoterapy, University Hospital Frankfurt, Goethe University, Frankfurt am Main, Germany; 26 Iriszorg Addiction Care, Nijmegen Institute for Scientist Practitioners in Addiction, Nijmegen, the Netherlands; 27 University Hospital of Psychiatry, Translational Research Center, University of Bern, Bern, Switzerland; 28 Department of Psychiatry, RadboudumcDonders Institute for Brain, Cognition and Behavior, Nijmegen, the Netherlands; 29 Nijmegen Institute for Scientist Practitioners in Addiction, Nijmegen, the Netherlands; 30 Südhang Clinic, Kirchlindach, Switzerland; 31 Mt. Sinai, Icahn School of Medicine, New York City, USA; 32 NYU Grossman School of Medicine, New York City, USA; 33 University of Applied Sciences-Utrecht, Utrecht, the Netherlands; 34 Amsterdam University Medical Centers, Location Academic Medical Center, Amsterdam, the Netherlands

**Keywords:** ADHD, SUD, Comorbidity

## Abstract

**Background:**

Substance use disorders (SUD) often co-occur with attention deficit hyperactivity disorder (ADHD). Although the short-term effects of some specific interventions have been investigated in randomized clinical trials, little is known about the long-term clinical course of treatment-seeking SUD patients with comorbid ADHD.

**Aims:**

This paper presents the protocol and baseline clinical characteristics of the International Naturalistic Cohort Study of ADHD and SUD (INCAS) designed and conducted by the International Collaboration on ADHD and Substance Abuse (ICASA) foundation. The overall aim of INCAS is to investigate the treatment modalities provided to treatment-seeking SUD patients with comorbid ADHD, and to describe the clinical course and identify predictors for treatment outcomes.

This ongoing study employs a multicentre observational prospective cohort design. Treatment-seeking adult SUD patients with comorbid ADHD are recruited, at 12 study sites in nine different countries. During the follow-up period of nine months, data is collected through patient files, interviews, and self-rating scales, targeting a broad range of cognitive and clinical symptom domains, at baseline, four weeks, three months and nine months.

**Results:**

A clinically representative sample of 578 patients (137 females, 441 males) was enrolled during the recruitment period (June 2017-May 2021). At baseline, the sample had a mean age (SD) of 36.7 years (11.0); 47.5% were inpatients and 52.5% outpatients; The most prevalent SUDs were with alcohol 54.2%, stimulants 43.6%, cannabis 33.1%, and opioids 14.5%. Patients reported previous treatments for SUD in 71.1% and for ADHD in 56.9%. Other comorbid mental disorders were present in 61.4% of the sample: major depression 31.5%, post-traumatic stress disorder 12.1%, borderline personality disorder 10.2%.

**Conclusions:**

The first baseline results of this international cohort study speak to its feasibility. Data show that many SUD patients with comorbid ADHD had never received treatment for their ADHD prior to enrolment in the study. Future reports on this study will identify the course and potential predictors for successful pharmaceutical and psychological treatment outcomes.

**Trial registration:**

ISRCTN15998989 20/12/2019.

## Introduction

Treatment-seeking patients with substance use disorders (SUD) are approximately three times more likely to meet criteria for attention-deficit/hyperactivity disorder (ADHD)the general population [1–4]. Studies have shown that SUD with comorbid ADHD, compared to SUD only, is associated with a more severe, chronic, and complex course of illness. This includes earlier onset of substance use [5, 6], a higher degree of poly-substance use [6, 7], more psychiatric comorbidity [8], chronicity [6, 7] and poorer SUD treatment outcomes [7, 9, 10]. Moreover, patients with SUD + ADHD have more psychosocial problems, higher number of care needs and higher rates of suicide-attempts than with SUD only [11, 12]. Finally, studies suggest that SUD + ADHD patients have more severe cognitive deficits than patients with ADHD only [13–17].

Although co-occurrence of ADHD in SUD patients has been associated with a poor prognosis of both conditions, little is known about the long-term course of treatment-seeking patients with SUD + ADHD, especially in routine clinical practice [9, 10, 18, 19]. The short-term efficacy of pharmacological treatment of patients with SUD + ADHD has been investigated in randomized controlled trials (RCT) with conflicting results [20]. A meta-analysis by Cunill et al. (2015) found that standard doses of ADHD medication may lead to a significant, but small reduction of core ADHD symptoms, with limited to no effects on substance use [10]. However, two later RCTs, not included in the meta-analysis by Cunill et al. (2015), that applied higher doses of extended-release mixed amphetamine salts or OROS methylphenidate did find a significant reduction of both ADHD symptoms and substance use, suggesting that higher doses of stimulants might be warranted in patients with comorbid stimulant use disorder and ADHD [21, 22]. Although stimulant treatment is not linked to the development of SUD among ADHD patients, and early childhood stimulant treatment may even prevent the development of adult SUD, the abuse potential of this class of medication has raised concerns regarding its safety for the treatment of patients with SUD + ADHD [23].

Few studies have investigated the efficacy of non-pharmacological treatments for patients with SUD + ADHD. Although cognitive behavioural therapy (CBT) has been shown to be effective in reducing ADHD symptom in patients with ADHD only [24], patients with comorbid SUD have been excluded from these studies. However, an RCT comparing integrated cognitive behavioural therapy (ICBT) with standard CBT was recently published and found that ICBT and CBT were equally effective in reducing substance use, while ICBT was more effective in reducing ADHD symptoms [25]. A qualitative study suggests that patients with ADHD + SUD wish for a coaching attitude and a dialectical behavioural therapy-based skills training has shown varying results [26, 27]. To the best of our knowledge, the effects of other non-pharmacological interventions, have not yet been investigated in ADHD + SUD.

Overall, despite evidence that pharmacological and non-pharmacological treatment should be provided to SUD individuals with comorbid ADHD, very little is known regarding the predictors for successful treatment outcomes in this debilitating condition. To fill this knowledge gap and inform future randomized trials, as well as clinicians and policymakers, the International Collaboration on ADHD and Substance Abuse (ICASA) foundation (www.adhdandsubstanceabuse.org) designed the International Naturalistic Cohort Study of ADHD and Substance Use Disorders Study (INCAS). The aims of this observational, multi-centre, longitudinal study is to describe the treatment modalities provided to individuals with SUD + ADHD entering SUD treatment, and to identify predictors of successful treatment outcomes, as measured by retention in treatment, the reduction in substance use and ADHD symptoms and the global functioning at three and nine-months follow-up. Furthermore, the safety profile of pharmacological treatments will be recorded. The results will be relevant to generate hypotheses for future treatment trials. The current paper describes the study protocol of this ongoing study and presents the baseline characteristics of the study participants.

## Methods

### Participants

Participants were recruited during the recruitment period (June 2017 – May 2021) at 12 different addiction treatment services in nine countries: Belgium, France, Germany, Hungary, the Netherlands, Spain, Sweden, Switzerland, and the USA. At each participating addiction treatment centre, patients (age ≥ 18 years) with moderate to severe SUD and with comorbid ADHD according to DSM-5, and thus meeting the inclusion criteria, were invited to participate at the start of a new treatment episode; defined as the first visit in the last three months or the first visit after receiving the diagnosis of adult ADHD. There were no formal exclusion criteria except incapability to complete the assessments.

As stated in the pre-registered study protocol available at https://doi.org/10.1186/ISRCTN15998989, we aimed to enrol 600 participants from 12 different sites in the following countries: Belgium, France, Germany, Hungary, the Netherlands, Spain, Sweden, Switzerland, and the USA. This study is expected to be finished in May 2022.

Participants received detailed written and oral study information before providing written informed consent. The study was approved by the Swedish Ethical Review Authority (2017/240–31) and is conducted in accordance with the Declaration of Helsinki – Ethical Principles for Medical Research Involving Humans Subjects. All participating study sites also received formal approval by their respective local medical ethical committees.

### Study design

This ongoing prospective international cohort study employs a naturalistic observational design in treatment seeking adult SUD patients with comorbid adult ADHD. During the follow-up period of nine months, data is collected through patient files, interviews and self-rating scales at admission to treatment (baseline), four weeks, three months and at nine months follow-up. Data entry is monitored by a central project coordinator monthly.

### Instruments

Assessment of ADHD, SUD and other comorbidities are done in accordance with local clinical routines and regulations. Information on the diagnostic procedure, e.g., the use of structured interviews, along with all other data collection, is entered and stored in a web-based electronic case report form (eCRF), provided by Clindox ®, which fits within the rules of regulation for Good Clinical Practice (GCP: European Medicines Agency, 2002) and the General Data Protection Regulation (GDPR). Information on provided treatment modalities, such as psychological and pharmacological treatments, including stimulant dosing, sociodemographic data (housing, employment, level of education etc.), age, gender, previous treatments, other psychiatric comorbidities, adverse events (in relation to ADHD medications) and misuse/diversion of prescriptive drugs was collected by clinicians at each site through patient interviews and patient files upon entering the data into the eCRF.

Treatment retention is defined as the number of days from the starting date of the new treatment episode until (premature) termination of treatment in accordance with or against the clinicians’ advice. Substance use during the 30 days before baseline and each follow-up visit, is quantified through the TimeLine FollowBack interview (TLFB: Sobell and Sobell 1992), counting the number of standard drinks per drinking day and defining a heavy drinking day as a day with at least five standard drinks (12 g alcohol/drink). Use of other substances is measured dichotomously, i.e., each day of use is coded “yes” or “no” respectively, regardless of the amount that was used on that day. Quantification of ADHD symptoms is performed at baseline and at each follow-up visit with the expanded version of the Adult ADHD Self-Report Scale (ASRS) [23, 24]. Overall global functioning is assessed by the clinician with the Clinical Global Impression Severity/Improvement scale (CGI-S/I) [25] and by the participant with the EuroQol-5D (EQ5D) [26].

In addition to the aforementioned predictors and outcome measures, seven self-rating scales are used at each assessment, to provide a refined dimensional architecture of ADHD + SUD and to identify potential mediators of treatment outcomes, including retention: 1) Self-efficacy through a 1-item question (“How confident are you that you will achieve the necessary behavioural change in the future – let’s say within the next 6 to 9 months?”). Based on the self-efficacy question, patients were also asked two additional questions, one on motivation for behavioural change related to substance use and one on the relevance of behavioural change related to substance use [28, 29]. 2) Craving through a 3-item questionnaire [30, 31], 3) anger and aggression through an 8-item questionnaire,4) sensitivity to reward through the 17-item sensitivity to reward scale of the shortened version of the Sensitivity to Punishment and Sensitivity to Reward Questionnaire [32], 5) severity of nicotine use through the 6-item version of the Fagerström Test for Nicotine Dependence [33], 6) emotion regulation through the 16-item short version of the Difficulties in Emotion Regulation Scale [34], and 7) religious salience through a 3-item questionnaire [35]..

### Statistical analysis

Analyses are mainly descriptive, describing the population at baseline and follow-up visits. Given the non-interventional observational design, the targeted sample size of enrolling 600 participants is not based on statistical power, but rather on the goal of providing a solid description of the specific patient population and variation in treatment modalities and clinical course.

In the current paper baseline characteristics are presented. In future publications additional analyses of baseline predictors, as independent variables of the main outcomes (retention to treatment, substance use as measured by TLFB, and ASRS score) as dependent variables, will be performed through survival analysis, regression models and mixed effect models. Other inferential statistical analyses will be considered using both univariate and multivariate models and considering the naturalistic (non-randomized) nature of the study using propensity score methods.

The power of the proposed analyses will depend on factors such as the number of treatment groups, the distribution of patients between the treatment groups, the differences in treatment effect between the different treatment modalities, and missing data. In the optimal case when there are only two treatment groups of interest (e.g., with and without pharmacotherapy), with approximately 300 patients per group, there will be approximately 80% power to detect a small to moderate effect size (Cohen’s d > 0.3) at a two-sided 5% significance level. However, less favourable distributions and possibly more treatment modalities may be found, and thus larger effect sizes are needed to be detectable.

## Results

Five hundred and seventy-eight participants, diagnosed with ADHD and comorbid SUD, were enrolled during the study period (June 2017-May 2021) at 12 addiction treatment centres in nine countries: Belgium (*n* = 65), France (*n* = 8), Germany (*n* = 56), Hungary (*n* = 15), the Netherlands (*n* = 72), Spain (*n* = 34), Sweden (*n* = 152), Switzerland (*n* = 135) and in the USA (*n* = 41). Participants received treatment either as inpatients (47.5%) or outpatients (52.5%).

The majority of the participants were male, and at enrolment to the study 53.8% were either unemployed or on sick leave. Mean age (SD) at baseline was 36.7 (11) years and the most prevalent SUDs were with alcohol, stimulants, cannabis and opioids. Most participants reported that they had received previous treatment for SUD and/or ADHD. Other comorbid psychiatric disorders were present in most cases, where the most common comorbidities were major depression, post-traumatic stress disorder and borderline personality disorder. Sociodemographic data is presented in Table 1, and clinical characteristics and information on treatment, at baseline, are presented in Tables 2 and 3, respectively.

**Table 1:** Sociodemographic data. Age is presented as mean with standard deviation

***N* = 578**	
**Age**	36.7 (11.0)
**Female/Male**	137/441 (23.7%/76.3%)
**Housing**	
Own accommodation	66.1%
Homeless	3.5%
Room	19.9%
Temporary housing	7.3%
Unknown	3.3%
Living alone	45.2%
Living with partner/parent/friend (or anyone)	52.9%
Unknown	1.9%
**Educational level**	
Not completed elementary school	2.1%
Completed elementary school	25.6%
Completed high school	38.9%
Completed tertiary education	30.4%
Unknown	2.9%
**Employment last 30 days**	
Student	4.3%
Income through work	40.3%
Unemployed/sick-leave	53.8%
Unknown	1.6%
Children below 18 years old in the household	
Yes	19.9%
No	77.3%
Unknown	2.8%

**Table 2:** Clinical characteristics. *N* = 578

**Age when ADHD diagnose was attained**	
0–12	16.3%
13–19	12.1%
≥ 20	69.2%
Unknown	2.4%
** ≥ 1 biological parent with SUD**	
Yes	44.8%
No	44.6%
Unknown	10.5%
**Moderate to severe SUD:**	
Alcohol	54.2%
Cannabis	33.2%
Hallucinogens	2.4%
Inhalants	3.1%
Opioids	14.5%
Stimulants	43.6%
Sedatives/hypnotics/anxiolytics	9.4%
Tobacco	63.5%
**Multiple SUDs (tobacco not included)**	
≥ 2 co-current SUDs (moderate to severe)	46.9%
≥ 3 co-current SUDs (moderate to severe)	14.0%
**Psychiatric co-morbidity (other than ADHD/SUD)**	
Yes	61.4%
No	36.0%
Unknown	2.6%
Antisocial personality disorder	2.2%
Anxiety disorders	5.5%
Autism spectrum disorder	6.1%
Bipolar and related disorders	4.3%
Borderline personality disorder	10.2%
Depressive disorders	31.5%
Disruptive, impulse-control, and conduct disorders	2.4%
Dissociative disorders	0.5%
Feeding and eating disorders	1.7%
Intellectual disability	2.2%
Motor disorder (e.g., Tourette's)	0.7%
Obsessive–compulsive and related disorders	2.1%
Schizophrenia spectrum and other psychotic disorders	1.7%
Somatic symptom and related disorders	1.2%
Trauma- and stressor-related disorders (e.g., PTSD)	12.1%

**Table 3:** Current and previous treatment for ADHD and SUD

**Previously received ADHD treatment**	
Yes	56.9%
No	40.8%
Unknown	2.3%
**Received pharmacological ADHD treatment before 18 years old**	
Yes	21.3%
No	51.0%
Unknown	27.6%
**Currently receives ADHD treatment (at inclusion)**	
Pharmacological	34.9%
Psychological	20.9%
No	60.7%
Unknown	1.7%
**Previously received SUD treatment**	
Yes	71.1%
No	24.0%
Unknown	4.9%

## Discussion

INCAS is a prospective observational cohort study, without prior funding, of treatment-seeking individuals with SUD and comorbid adult ADHD, designed to describe the clinical course in different treatment settings and countries. It will provide a rich data set on the treatment modalities offered, the clinical course of the study population, and the potential influence of age, gender, primary substance of abuse, and other psychiatric comorbidities on treatment outcomes. Moreover, the study includes longitudinal data on a broad range of psychological measures, targeting a diverse set of cognitive domains and symptoms, including core deficits seen in ADHD and SUD.

In our sample there is an expected male-to-female ratio of approximately 3:1, although sex differences in SUD and ADHD are poorly understood [36]. Given the large sample size in the present study, including a variety of detailed longitudinal data, this dataset provides an opportunity to explore sex differences in SUD patients with comorbid ADHD, and to analyse the association with treatment outcome measures. A large proportion (40.8%) reported that they had not previously received any ADHD treatment prior to enrolment in our study, while most (71.1%) had previously received SUD treatment. This speaks to the importance of screening for ADHD in SUD treatment-seeking patients. Furthermore, our sample partly consists of participants with a severe and complex psychiatric burden, where a majority presented with at least one other psychiatric comorbidity (61.4%), and almost half our sample (46.9%) were diagnosed with two or more co-current SUDs. This will allow us to explore subgroups within this population and possibly identify important predictors for treatment outcomes.

Overall, the presented overview of the baseline characteristics, reveals that our sample consists of participants with a diverse distribution of socio-economic, psychosocial, and educational backgrounds. This will improve inference and serve to the generalizability of our findings. In addition, our sample consists of patients with different clinical backgrounds, psychiatric co-morbidities, and severity levels, where symptoms and severity of symptoms is collected with a high level of detail.

The main strengths of this study are the naturalistic design and the related ecological validity, the use of an identical study design with the same assessments at each treatment facility in various countries, and the large sample size allowing subgroup analyses and comparisons. However, the naturalistic design is also one of the most important limitations. For instance, the treatments are not blinded nor randomly allocated, thus causing inferential challenges and difficulties in controlling for selection and information bias and/or confounding. Finally, a limitation is that different sites vary in diagnostic and treatment procedures. The main purpose of the study is, however, descriptive and aims to describe this specific patient population and the treatments provided to them. The study will provide information on treatment effects and the clinical course of these disorders with simultaneously collected data on substance use, ADHD-symptoms, and quality of life.

Data analysis will consider limitations, including the use of propensity scores to control for baseline differences between groups. Moreover, given the high number of patients and the level of detail in which data is collected, it will be possible to analyse associations and provide insight on treatment effects on a broad range of treatment modalities and outcomes. Possible statistical inferential challenges of the findings will be further discussed in subsequent papers.

In conclusion, these first results speak to the feasibility of the study, despite no prior funding, and will provide an overall representative, description of the characteristics and the clinical course of treatment-seeking patients with SUD and comorbid adult ADHD at the international level. In addition, this study will provide information on potential predictors for successful treatment outcomes for different treatment modalities and thus hypotheses for future randomized controlled trials.

## Data Availability

The datasets used and/or analysed during the current study are available from the corresponding author on reasonable request.

## Abbreviations

SUD: Substance Use Disorder
ADHD: Attention Deficit/Hyperactivity Disorder
INCAS: International Naturalistic Cohort Study of ADHD and SUD
ICASA: International Collaboration on ADHD and Substance Abuse
RCT: Randomized Controlled Trial
CBT: Cognitive Behavioural Therapy
ICBT: Integrated Cognitive Behavioural Therapy
DSM-5: Diagnostic and Statistical Manual of Mental Disorders
eCRF: Electronic Case Report Form
TLFB: Time-Line Follow-Back
ASRS: Adult ADHD Self-Report Scale

## References

[1] Charach A, Yeung E, Climans T, Lillie E (2011). Childhood Attention-Deficit/Hyperactivity Disorder and Future Substance Use Disorders: Comparative Meta-Analyses. J Am Acad Child Adolesc Psychiatry.

[2] Kessler R (2006). The prevalence and correlates of adult ADHD in the United States: results from the National Comorbidity Survey Replication. Am J Psychiatr.

[3] van de Glind G, Konstenius M, Koeter MWJ, van Emmerik-van OK, Carpentier PJ, Kaye S (2014). Variability in the prevalence of adult ADHD in treatment seeking substance use disorder patients: results from an international multi-center study exploring DSM-IV and DSM-5 criteria. Drug Alcohol Depend.

[4] van Emmerik-van OK, van de Glind G, van den Brink W, Smit F, Crunelle CL, Swets M (2012). Prevalence of attention-deficit hyperactivity disorder in substance use disorder patients: a meta-analysis and meta-regression analysis. Drug Alcohol Depend.

[5] Kaye S, Ramos-Quiroga JA, van de Glind G, Levin FR, Faraone SV, Allsop S (2019). Persistence and Subtype Stability of ADHD Among Substance Use Disorder Treatment Seekers. J Atten Disord.

[6] Fatseas M, Hurmic H, Serre F, Debrabant R, Daulouede JP, Denis C (2016). Addiction severity pattern associated with adult and childhood Attention Deficit Hyperactivity Disorder (ADHD) in patients with addictions. Psychiatry Res.

[7] Young JT, Carruthers S, Kaye S, Allsop S, Gilsenan J, Degenhardt L (2015). Comorbid attention deficit hyperactivity disorder and substance use disorder complexity and chronicity in treatment-seeking adults. Drug Alcohol Rev.

[8] van Emmerik-van OK, van de Glind G, Koeter MW, Allsop S, Auriacombe M, Barta C (2014). Psychiatric comorbidity in treatment-seeking substance use disorder patients with and without attention deficit hyperactivity disorder: results of the IASP study. Addiction.

[9] Levin FR, Evans SM, Vosburg SK, Horton T, Brooks D, Ng J (2004). Impact of attention-deficit hyperactivity disorder and other psychopathology on treatment retention among cocaine abusers in a therapeutic community. Addict Behav.

[10] Cunill R, Castells X, Tobias A, Capella D (2015). Pharmacological treatment of attention deficit hyperactivity disorder with co-morbid drug dependence. J Psychopharmacol.

[11] Kronenberg LM, Goossens PJ, van Etten DM, van Achterberg T, van den Brink W (2015). Need for care and life satisfaction in adult substance use disorder patients with and without attention deficit hyperactivity disorder (ADHD) or autism spectrum disorder (ASD). Perspect Psychiatr Care.

[12] Rodriguez-Cintas L, Daigre C, Braquehais MD, Palma-Alvarez RF, Grau-Lopez L, Ros-Cucurull E (2018). Factors associated with lifetime suicidal ideation and suicide attempts in outpatients with substance use disorders. Psychiatry Res.

[13] Vonmoos M, Hulka LM, Preller KH, Jenni D, Baumgartner MR, Stohler R (2013). Cognitive dysfunctions in recreational and dependent cocaine users: role of attention-deficit hyperactivity disorder, craving and early age at onset. Br J Psychiatry.

[14] Brooks DJ, Vosburg SK, Evans SM, Levin FR (2006). Assessment of cognitive functioning of methadone-maintenance patients: impact of adult ADHD and current cocaine dependence. J Addict Dis.

[15] Duarte NA, Woods SP, Rooney A, Atkinson JH, Grant I, Translational Methamphetamine ARCG (2012). Working memory deficits affect risky decision-making in methamphetamine users with attention-deficit/hyperactivity disorder. J Psychiatr Res.

[16] Crunelle CL, Veltman DJ, van Emmerik-van OK, Booij J, van den Brink W (2013). Impulsivity in adult ADHD patients with and without cocaine dependence. Drug Alcohol Depend.

[17] Miguel CS, Martins PA, Moleda N, Klein M, Chaim-Avancini T, Gobbo MA (2016). Cognition and impulsivity in adults with attention deficit hyperactivity disorder with and without cocaine and/or crack dependence. Drug Alcohol Depend.

[18] Crunelle CL, van den Brink W, Moggi F, Konstenius M, Franck J, Levin FR (2018). International Consensus Statement on Screening, Diagnosis and Treatment of Substance Use Disorder Patients with Comorbid Attention Deficit/Hyperactivity Disorder. Eur Addict Res.

[19] Kooij JJS, Bijlenga D, Salerno L, Jaeschke R, Bitter I, Balazs J (2019). Updated European Consensus Statement on diagnosis and treatment of adult ADHD. Eur Psychiatry.

[20] Carpentier PJ, Levin FR (2017). Pharmacological Treatment of ADHD in Addicted Patients: What Does the Literature Tell Us?. Harv Rev Psychiatry.

[21] Levin FR, Mariani JJ, Specker S, Mooney M, Mahony A, Brooks DJ (2015). Extended-Release Mixed Amphetamine Salts vs Placebo for Comorbid Adult Attention-Deficit/Hyperactivity Disorder and Cocaine Use Disorder: A Randomized Clinical Trial. JAMA Psychiat.

[22] Konstenius M, Jayaram-Lindstrom N, Guterstam J, Beck O, Philips B, Franck J (2014). Methylphenidate for attention deficit hyperactivity disorder and drug relapse in criminal offenders with substance dependence: a 24-week randomized placebo-controlled trial. Addiction.

[23] Faraone SV, Wilens TE. Effect of Stimulant Medications for Attention-Deficit_Hyperactivity Disorder on Later Substance Use and the Potential for Stimulant Misuse, Abuse, and Diversion. J Clin Psychiatr. 2007;2007;68:11:5–22. 18307377

[24] National Collaborating Centre for Mental H. National Institute for Health and Clinical Excellence: Guidance. Attention Deficit Hyperactivity Disorder: Diagnosis and Management of ADHD in Children, Young People and Adults. Leicester (UK): British Psychological Society (UK). Copyright © 2009, The British Psychological Society & The Royal College of Psychiatrists.; 2009.

[25] van Emmerik-van OK, Vedel E, Kramer FJ, Blankers M, Dekker JJM, van den Brink W (2019). Integrated cognitive behavioral therapy for ADHD in adult substance use disorder patients: Results of a randomized clinical trial. Drug Alcohol Depend.

[26] Bihlar Muld B, Jokinen J, Bolte S, Hirvikoski T (2016). Skills training groups for men with ADHD in compulsory care due to substance use disorder: a feasibility study. Atten Defic Hyperact Disord.

[27] Kronenberg LM, Verkerk-Tamminga R, Goossens PJ, van den Brink W, van Achterberg T (2015). Personal recovery in individuals diagnosed with substance use disorder (SUD) and co-occurring attention deficit/hyperactivity disorder (ADHD) or autism spectrum disorder (ASD). Arch Psychiatr Nurs.

[28] Ilgen M, McKellar J, Tiet Q (2005). Abstinence self-efficacy and abstinence 1 year after substance use disorder treatment. J Consult Clin Psychol.

[29] Ludwig F, Tadayon-Manssuri E, Strik W, Moggi F (2013). Self-efficacy as a predictor of outcome after residential treatment programs for alcohol dependence: simply ask the patient one question!. Alcohol Clin Exp Res.

[30] Nakovics H, Diehl A, Geiselhart H, Mann K (2009). Development and validation of an overall instrument to measure craving across multiple substances: the Mannheimer Craving Scale (MaCS). Psychiatr Prax.

[31] Reichenbach F, Burren Y, Flückiger C, Znoj H, Moggi F (2018). Swiss Study to Validate the Mannheimer Craving Scale (MaCS). Sucht.

[32] O’Connor RM, Colder CR, Hawk LW (2004). Confirmatory factor analysis of the Sensitivity to Punishment and Sensitivity to Reward Questionnaire. Personality Individ Differ.

[33] Heatherton TF, Kozlowski LT, Frecker RC, Fagerström K-O (1991). The Fagerstrom Test for Nicotine Dependence: a revision of the Fagerstrom Tolerance Questionnaire. Br J Addict.

[34] Bjureberg J, Ljotsson B, Tull MT, Hedman E, Sahlin H, Lundh LG (2016). Development and Validation of a Brief Version of the Difficulties in Emotion Regulation Scale: The DERS-16. J Psychopathol Behav Assess.

[35] Koenig HG, Büssing A (2010). The Duke University Religion Index (DUREL): A Five-Item Measure for Use in Epidemological Studies. Religions (Basel, Switzerland ).

[36] Slobodin O, Davidovitch M (2019). Gender Differences in Objective and Subjective Measures of ADHD Among Clinic-Referred Children. Front Hum Neurosci.

